# Supplementary material for: More Sophisticated Is Not Always Better: A Comparison of Similarity Measures for Unsupervised Learning of Pathways in Biomolecular Simulations
Source: J Phys Chem B. 2025 Oct 8;129(42):10956–66. doi: 10.1021/acs.jpcb.5c04586 (PMC12557397; doi:10.1021/acs.jpcb.5c04586)
Supplement: Supplementary file 1 [file jp5c04586_si_001.pdf]

Supporting Information for Publication: More Sophisticated is Not Always Better: A Comparison of Similarity Measures for Unsupervised Learning of Pathways in Biomolecular Simulations

Miriam Jäger[1] and Steffen Wolf*[1]
Biomolecular Dynamics, Institute of Physics, University of Freiburg, 79104 Freiburg, Germany [a)]

(*Electronic mail: steffen.wolf@physik.uni-freiburg.de.)

S1. STREPTAVIDIN-BIOTIN SIMULATION DETAILS

The simulations were carried out using different pulling vectors in space with a Cartesian (x,y,z) nomenclature. For each pulling direction, 50 restraint simulations and 200 constraint simulations were started, respectively. Table S1 shows the pulling directions with the corresponding number of successful trajectories. Unsuccessful trajectories resulted from pulling the ligand unphysically into the protein and causing a simulation "crash" due to too high inter-particle forces.

**Pulling Vector**	Number of trajectories	
	constraint	restraint
(1, 0, 1)	71	40
(1, 1, 0)	93	50
(1, -1, 0)	142	48

TABLE S1: Number of successful trajectories for each pulling vector in constraint and restraint simulations.

S2. PERFORMANCE OF SIMILARITY MEASURES

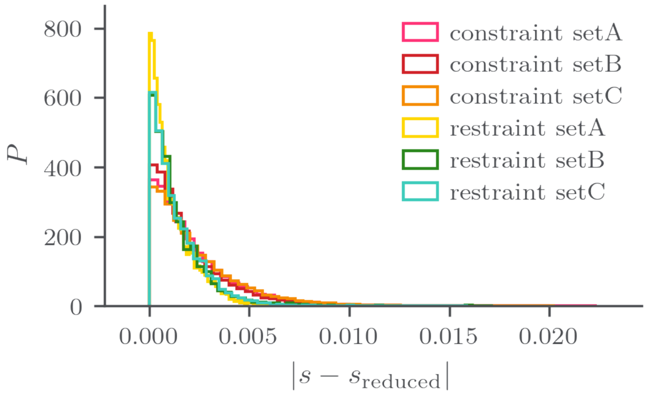

FIG. S1: Distribution of the absolute difference between the similarities calculated from the full trajectories (s) and trajectories downsampled to every 5th time step (s_{reduced}) of the different streptavidin-biotin test sets.

[a)]https://www.moldyn.uni-freiburg.de/

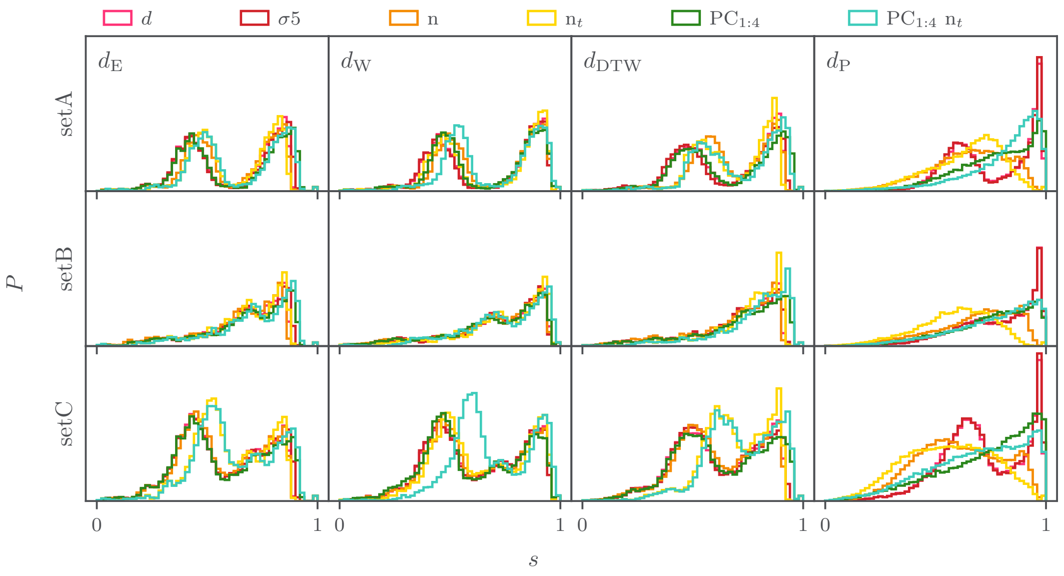

FIG. S2: Comparison of the influence of preprocessing on the similarity distribution for streptavidin-biotin constraint simulations sets A, B and C. The similarity measure is given by the label inside the Figure. The data preprocessing is given as color code in the legend.

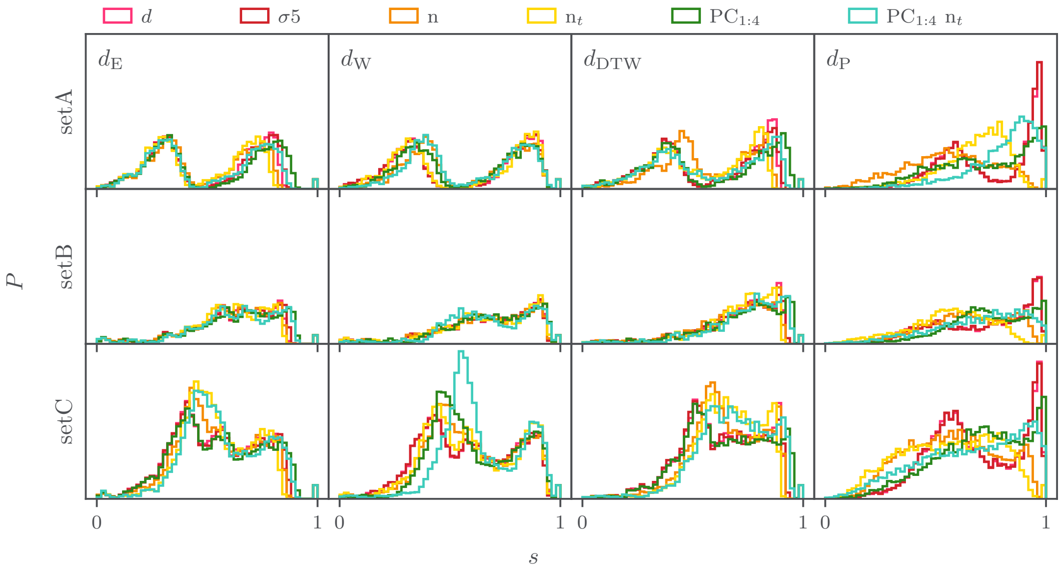

FIG. S3: Comparison of the influence of preprocessing on the similarity distribution for streptavidin-biotin restraint simulations sets A, B and C. The similarity measure is given by the label inside the Figure. The data preprocessing is given as color code in the legend.

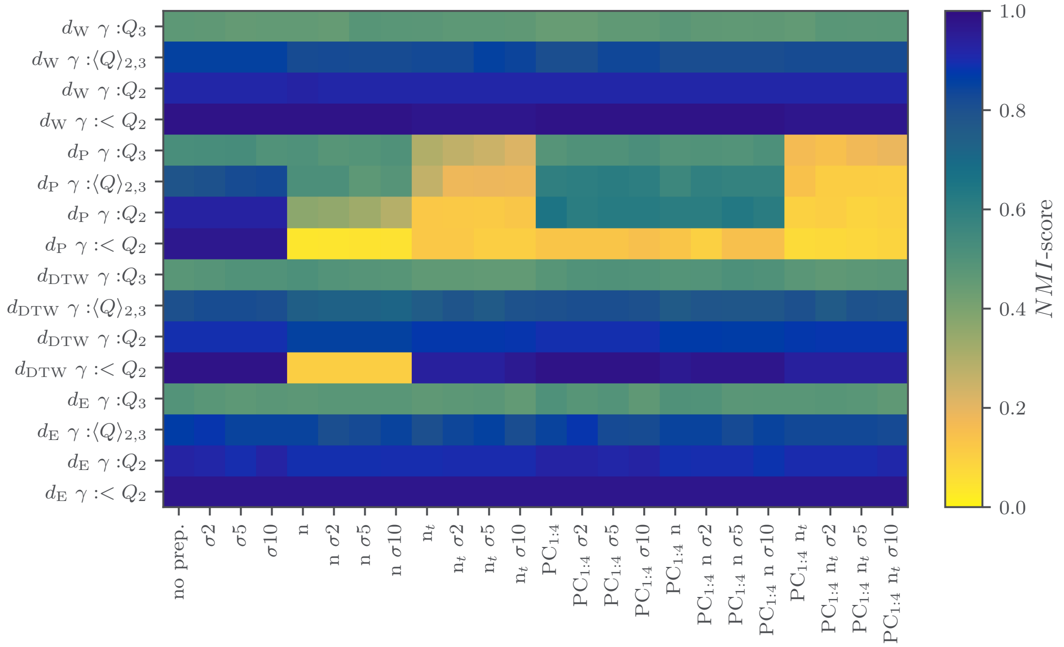

FIG. S4: Comparison of clustering results from streptavidin-biotin constraint simulations set A with ground truth clusters using the NMI score. The y-axis indicates the similarity measure used and the resolution parameter γ: Q_2 represents the median of the similarity distribution, Q_3 the third quartile, $\langle Q \rangle_{2,3}$ the mean of Q_2 and Q_3, and $< Q_2$ denotes $Q_2 - 0.1$. The x-axis indicates the preprocessing applied to the dataset before similarity calculation: σ specifies the Gaussian filter width, n denotes global normalization, n_t indicates time-resolved normalization and $PC_{1:4}$ refers to PCs 1-4 were used.

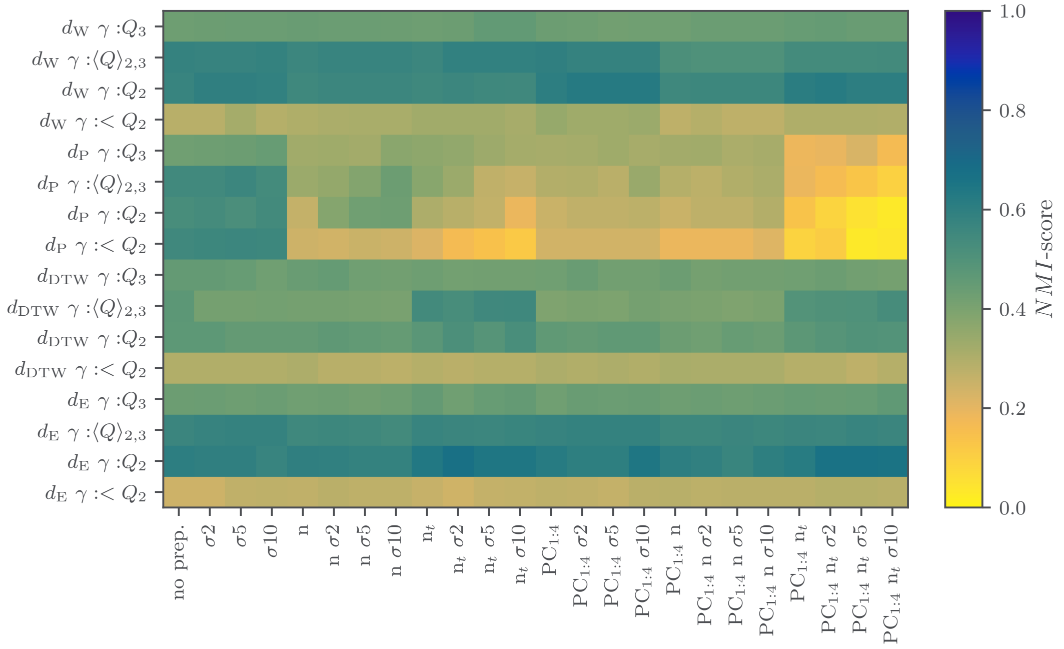

FIG. S5: Comparison of clustering results from streptavidin-biotin constraint simulations set B with ground truth clusters using the NMI score. The y-axis indicates the similarity measure used and the resolution parameter γ: Q_2 represents the median of the similarity distribution, Q_3 the third quartile, $\langle Q \rangle_{2,3}$ the mean of Q_2 and Q_3 and $< Q_2$ denotes $Q_2 - 0.1$. The x-axis indicates the preprocessing applied to the dataset before similarity calculation: σ specifies the Gaussian filter width, n denotes global normalization, n_t indicates time-resolved normalization and $PC_{1:4}$ refers to PCs 1-4 were used.

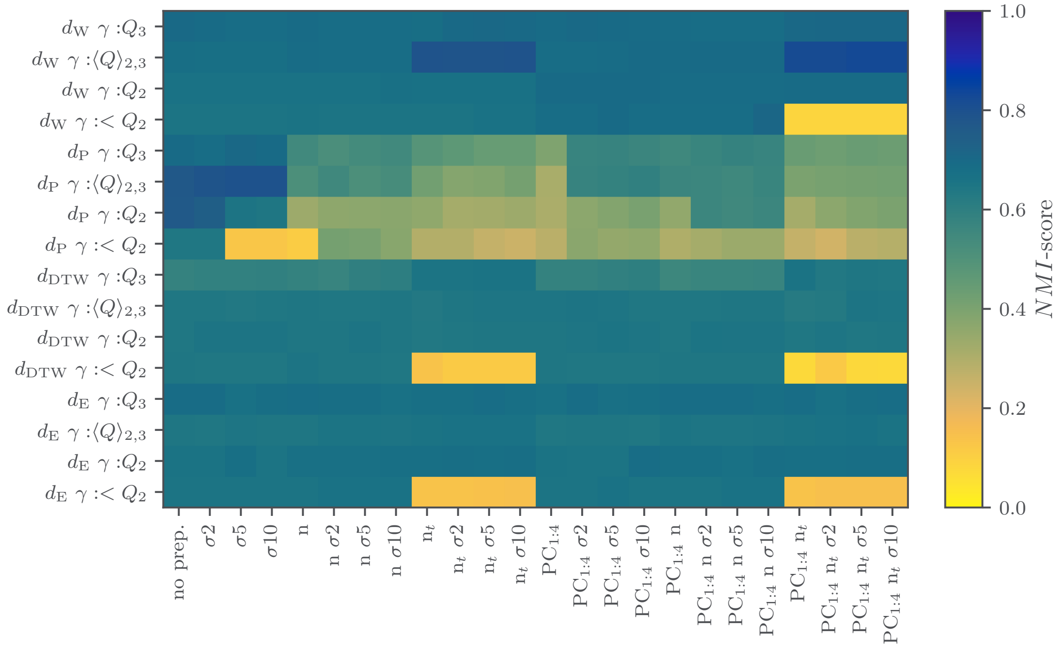

FIG. S6: Comparison of clustering results from streptavidin-biotin constraint simulations set C with ground truth clusters using the NMI score. The y-axis indicates the similarity measure used and the resolution parameter γ: Q_2 represents the median of the similarity distribution, Q_3 the third quartile, $\langle Q \rangle_{2,3}$ the mean of Q_2 and Q_3, and $< Q_2$ denotes $Q_2 - 0.1$. The x-axis indicates the preprocessing applied to the dataset before similarity calculation: σ specifies the Gaussian filter width, n denotes global normalization, n_t indicates time-resolved normalization and $PC_{1:4}$ refers to PCs 1-4 were used.

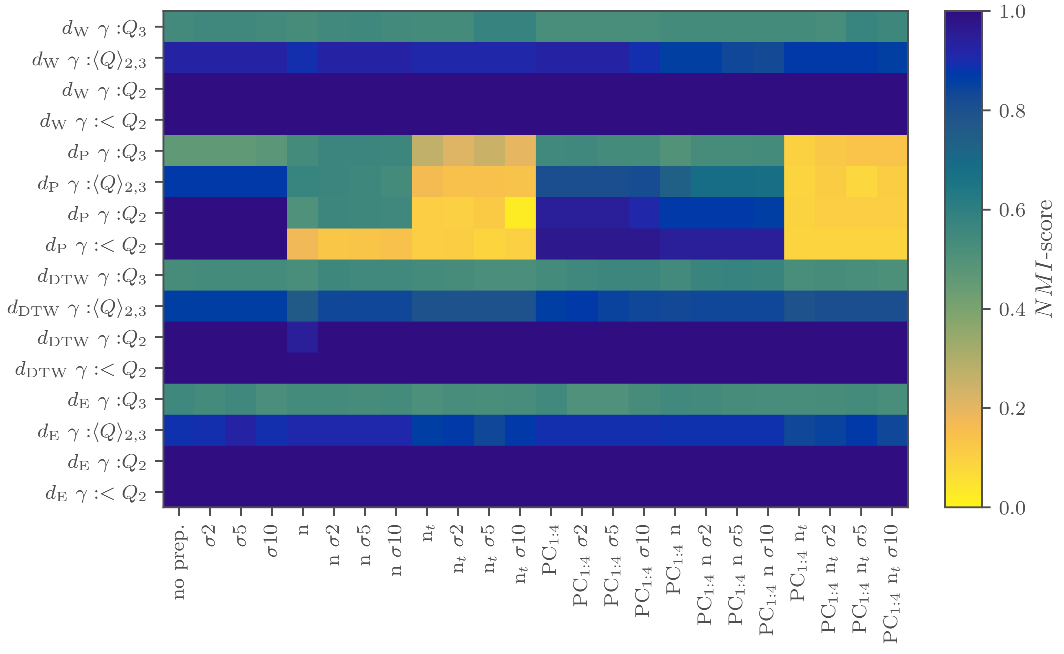

FIG. S7: Comparison of clustering results from streptavidin-biotin restraint simulations set A with ground truth clusters using the NMI score. The y-axis indicates the similarity measure used and the resolution parameter γ: Q_2 represents the median of the similarity distribution, Q_3 the third quartile, $\langle Q \rangle_{2,3}$ the mean of Q_2 and Q_3, and $< Q_2$ denotes $Q_2 - 0.1$. The x-axis indicates the preprocessing applied to the dataset before similarity calculation: σ specifies the Gaussian filter width, n denotes global normalization, n_t indicates time-resolved normalization and $PC_{1:4}$ refers to PCs 1-4 were used.

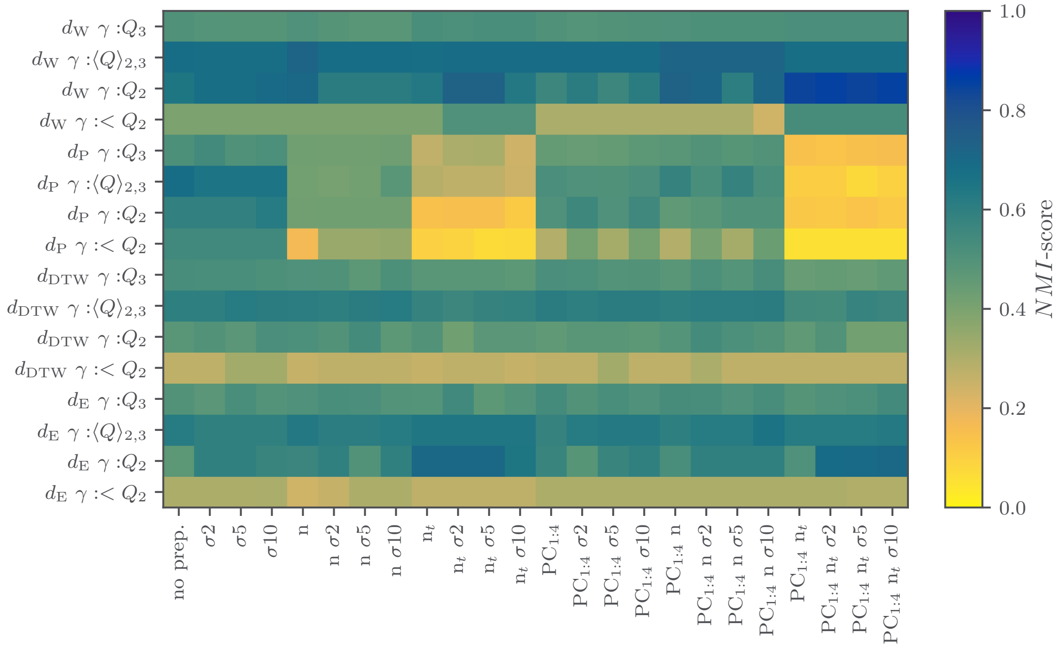

FIG. S8: Comparison of clustering results from streptavidin-biotin restraint simulations set B with ground truth clusters using the NMI score. The y-axis indicates the similarity measure used and the resolution parameter γ: Q_2 represents the median of the similarity distribution, Q_3 the third quartile, $\langle Q\rangle_{2,3}$ the mean of Q_2 and Q_3, and $< Q_2$ denotes $Q_2 - 0.1$. The x-axis indicates the preprocessing applied to the dataset before similarity calculation: σ specifies the Gaussian filter width, n denotes global normalization, n_t indicates time-resolved normalization and $PC_{1:4}$ refers to PCs 1-4 were used.

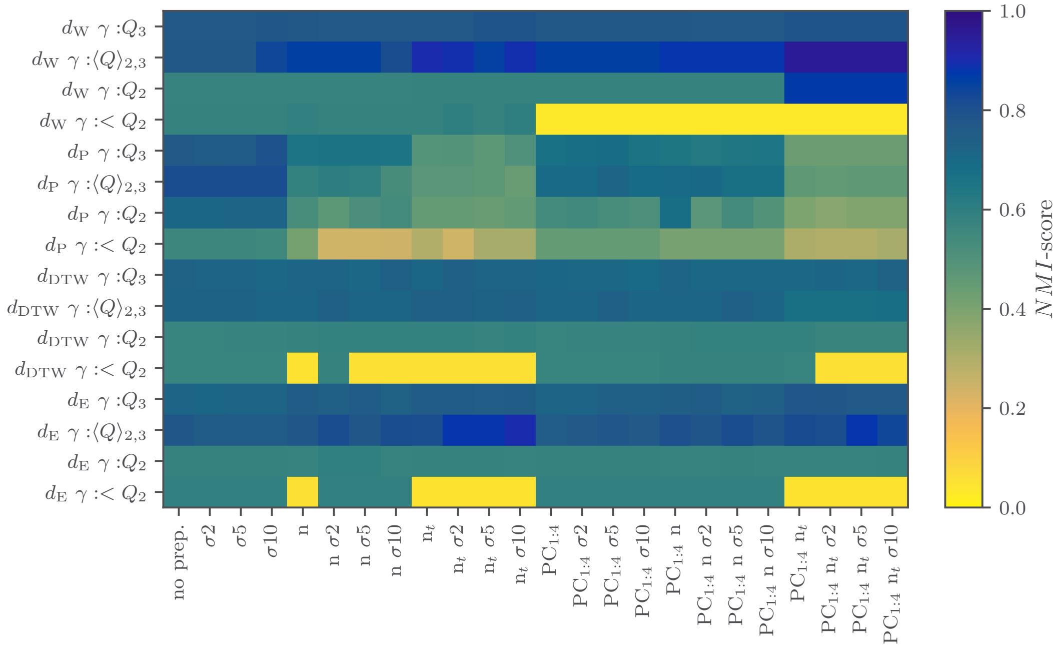

FIG. S9: Comparison of clustering results from streptavidin-biotin restraint simulations set C with ground truth clusters using the NMI score. The y-axis indicates the similarity measure used and the resolution parameter γ: Q_2 represents the median of the similarity distribution, Q_3 the third quartile, $\langle Q\rangle_{2,3}$ the mean of Q_2 and Q_3, and $< Q_2$ denotes $Q_2 - 0.1$. The x-axis indicates the preprocessing applied to the dataset before similarity calculation: σ specifies the Gaussian filter width, n denotes global normalization, n_t indicates time-resolved normalization and $PC_{1:4}$ refers to PCs 1-4 were used.

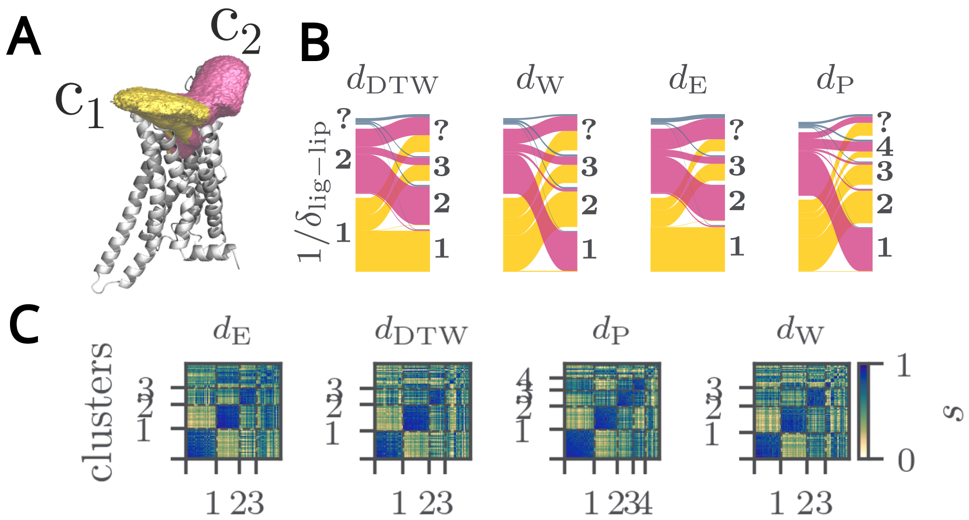

FIG. S10: Clustering results for the A_{2a} adenosine receptor-inhibitor complex using $\gamma = Q_3$ compared to known clustering results based on the inverse minimal ligand-lipid distance $1/\delta_{\text{lig-lip}}$ as a reaction coordinate with microscopically feasible unbinding mechanism. A: Visualization of cluster 1 and 2 as volumes based on inverse ligand lipid distances B: Sankey diagram comparing geometrical path separation results with $1/\delta_{\text{lig-lip}}$ clustered with $\gamma = Q_2$. C: Corresponding block-ordered similarity matrix.

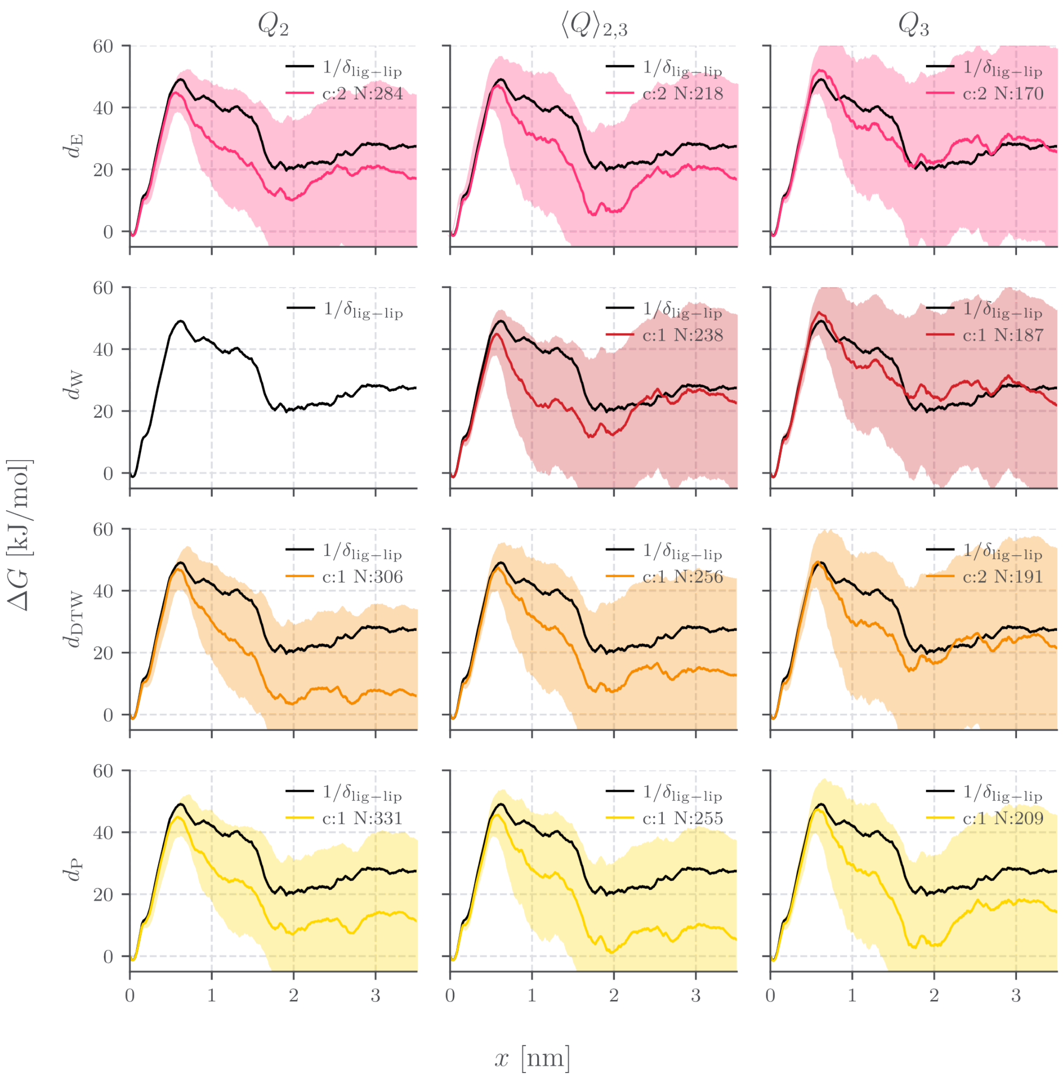

FIG. S11: dcTMD results of different A_{2a} clusters. The shaded area signifies the bootstrapping errors from 5000 resamples in a 90% confidence interval.

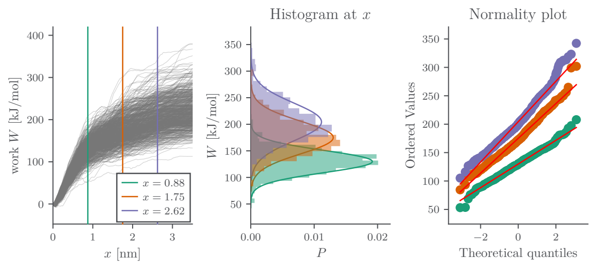

(a) all trajectories

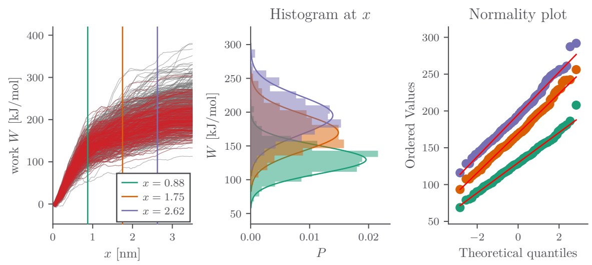

(b) $1/\delta_{\text{lig-lip}}$ c_2 $\gamma = Q_2$

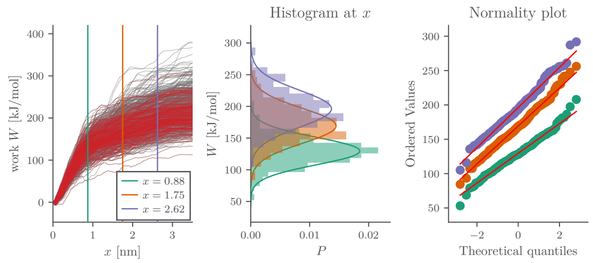

(c) d_{E} c_2 $\gamma = Q_2$

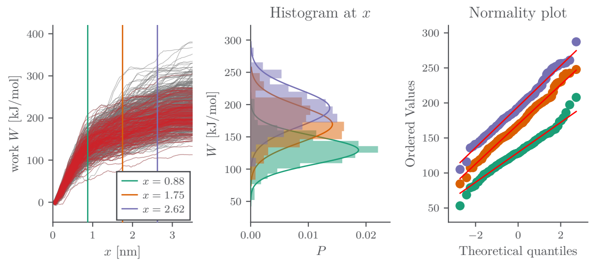

(d) d_{E} c_2 $\gamma = \langle Q \rangle_{23}$

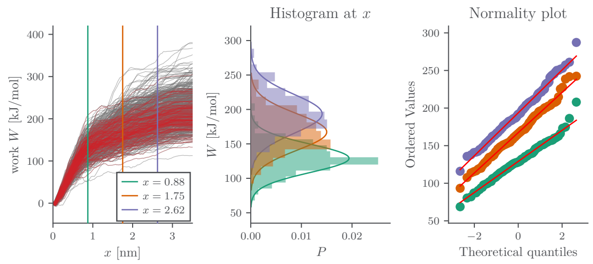

(e) d_{E} c_2 $\gamma = Q_3$

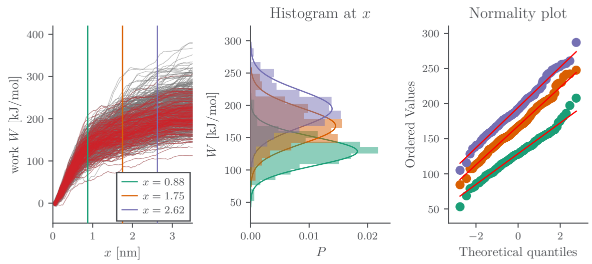

(f) d_{W} c_1 $\gamma = \langle Q \rangle_{23}$

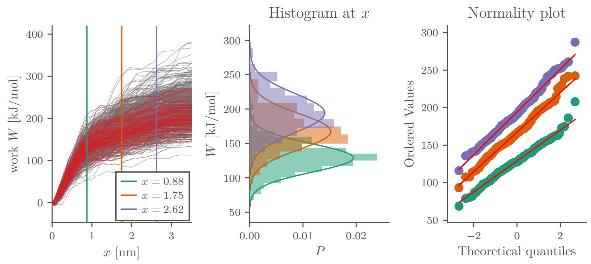

(g) d_{W} c_1 $\gamma = Q_3$

FIG. S12: Work distribution analysis of clusters in Fig. S11. The similarity measure, cluster index and used γ is indicated in the subcaptions. Left: Work values of all trajectories in gray, overlaid by the trajectories in the respective cluster in red. Middle: Histograms at values of x indicated in the left plot. Right: Corresponding QQ plots.

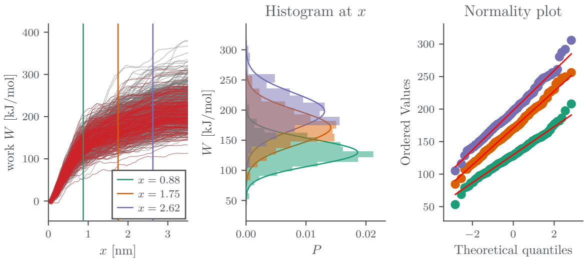

(a) d_{DTW} c_1 $\gamma = Q_2$

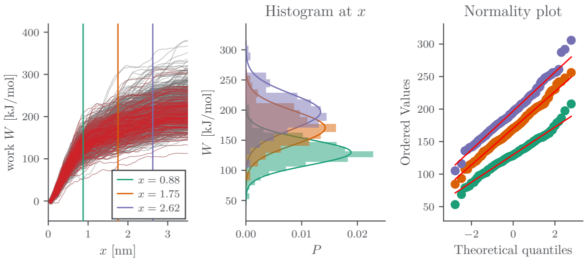

(b) d_{DTW} c_1 $\gamma = \langle Q \rangle_{23}$

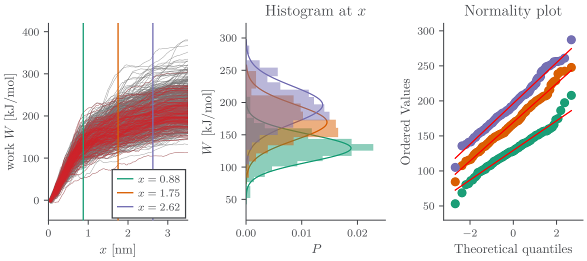

(c) d_{DTW} c_2 $\gamma = Q_3$

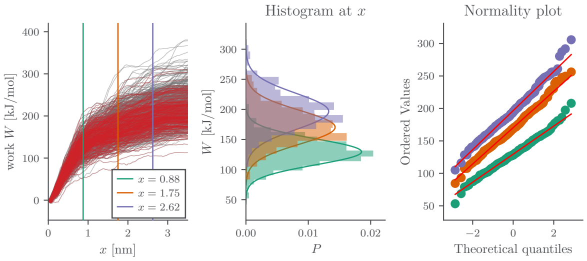

(d) d_{P} c_1 $\gamma = Q_2$

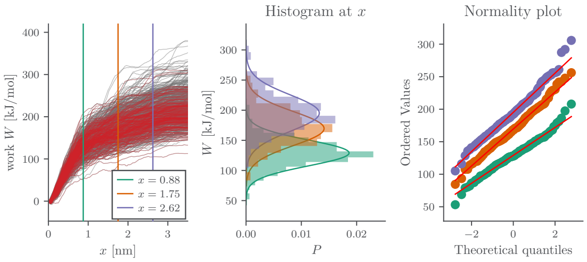

(e) d_{P} c_1 $\gamma = \langle Q \rangle_{23}$

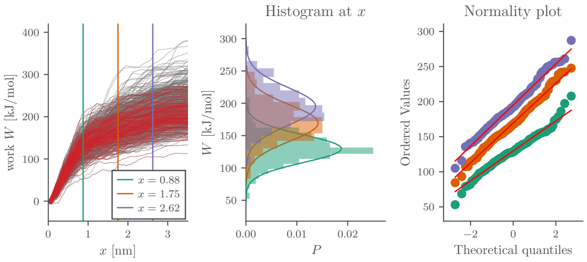

(f) d_{P} c_1 $\gamma = Q_3$

FIG. S13: Work distribution analysis of clusters in Fig. S11. The similarity measure, cluster index and used γ is indicated in the subcaptions. Left: Work values of all trajectories in gray, overlaid by the trajectories in the respective cluster in red. Middle: Histograms at values of x indicated in the left plot. Right: Corresponding QQ plots.
